# Multiple Small RNAs Interact to Co-regulate Ethanol Tolerance in *Zymomonas mobilis*

**DOI:** 10.3389/fbioe.2020.00155

**Published:** 2020-03-04

**Authors:** Runhua Han, Katie Haning, Juan C. Gonzalez-Rivera, Yongfu Yang, Runxia Li, Seung Hee Cho, Ju Huang, Bobi A. Simonsen, Shihui Yang, Lydia M. Contreras

**Affiliations:** ^1^McKetta Department of Chemical Engineering, The University of Texas at Austin, Austin, TX, United States; ^2^State Key Laboratory of Biocatalysis and Enzyme Engineering, Hubei Collaborative Innovation Center for Green Transformation of Bio-resources, Environmental Microbial Technology Center of Hubei Province, and School of Life Sciences, Hubei University, Wuhan, China; ^3^Institute for Cellular and Molecular Biology, College of Natural Sciences, The University of Texas at Austin, Austin, TX, United States

**Keywords:** *Zymomonas mobilis*, small RNA, sRNA interactions, integrated omics, ethanol tolerance

## Abstract

sRNAs represent a powerful class of regulators that influences multiple mRNA targets in response to environmental changes. However, very few direct sRNA–sRNA interactions have been deeply studied in any organism. *Zymomonas mobilis* is a bacterium with unique ethanol-producing metabolic pathways in which multiple small RNAs (sRNAs) have recently been identified, some of which show differential expression in ethanol stress. In this study, we show that two sRNAs (Zms4 and Zms6) are upregulated under ethanol stress and have significant impacts on ethanol tolerance and production in *Z. mobilis*. We conducted multi-omics analysis (combining transcriptomics and sRNA-immunoprecipitation) to map gene networks under the influence of their regulation. We confirmed that Zms4 and Zms6 bind multiple RNA targets and regulate their expressions, influencing many downstream pathways important to ethanol tolerance and production. In particular, Zms4 and Zms6 interact with each other as well as many other sRNAs, forming a novel sRNA–sRNA direct interaction network. This study thus uncovers a sRNA network that co-orchestrates multiple ethanol related pathways through a diverse set of mRNA targets and a large number of sRNAs. To our knowledge, this study represents one of the largest sRNA–sRNA direct interactions uncovered so far.

## Introduction

As global controllers of gene expression, small RNAs (sRNAs) represent powerful tools for engineering complex phenotypes (Cho et al., 2015; Leistra et al., 2019). These (typically) non-coding RNAs are 5–500 nucleotide (nt) transcripts that act as regulators of protein expression, mostly by blocking translation or changing mRNA stability (Storz et al., 2011). Although less common, proteins have also been shown to be targets of sRNA regulation (Pichon and Felden, 2007). Traditionally thought of as non-coding RNAs, many have been discovered in intergenic regions (Tsai et al., 2015), although some are now known to produce small peptides (Pichon and Felden, 2007). The majority of well-studied sRNAs act in *trans*, meaning their targets are encoded elsewhere in the genome; this is in contrast to *cis*-encoded sRNAs, which neighbor their targets on the same or opposite DNA strand (Gottesman and Storz, 2011).

Advances in high-throughput sequencing have enabled discovery of hundreds of sRNAs across bacteria (Tsai et al., 2015; Hor et al., 2018), but characterization lags far behind. As a result, the vast majority of sRNAs have functions completely unknown, especially in non-model organisms. Current approaches take advantage of RNA-seq and proteomics to determine sRNA target networks (Lalaouna and Masse, 2015; Melamed et al., 2016), although a challenge remains to decouple direct vs. indirect interactions. Computational tools such as IntaRNA can be helpful in predicting most favorable sRNA–mRNA interactions and binding sites, although *in vivo* conditions and competition of multiple targets for binding sites cannot be accounted for (Busch et al., 2008). Ultimately, electrophoretic mobility shift assays (EMSAs) and reporter gene systems can complement these approaches in testing direct binding of RNA and protein targets *in vitro* and *in vivo* (Corcoran et al., 2012; Tomasini et al., 2017). Most recent, the mapping of sRNA interfaces that could be available *in vivo* for interactions has also been useful to determine biologically relevant mRNA targets (Vazquez-Anderson et al., 2017; Mihailovic et al., 2018).

Increasingly complex regulatory networks have been discovered, including several direct sRNA–sRNA interactions (Vogel et al., 2003; Lybecker et al., 2014; Miyakoshi et al., 2015; Frohlich et al., 2016). One reported interaction is between sRNAs SraC and SdsR in *Escherichia coli*, which originates from the same intergenic region, encoded in opposite directions (Vogel et al., 2003). However, due to the complete overlap of SdsR in antisense sequence to SraC, the binding of these sRNAs is expected and it results in RNase III-dependent cleaving (Vogel et al., 2003). Although still largely uncharacterized in *E. coli*, the target network of SdsR has been characterized in *Salmonella enterica* and includes stress response regulators (Frohlich et al., 2016). Another known interaction is between sRNA GcvB and the RNA sponge SroC, which represses GcvB in *E. coli* (Miyakoshi et al., 2015). This mRNA cross-talk forms a feed-forward loop in the regulation of ABC transporters and affects growth in different nutrient conditions (Miyakoshi et al., 2015). Additionally, two sRNAs (AsxR and AgvB) have been identified within bacteriophage-derived regions in enterohemorrhagic *E. coli* acting as “anti-sRNAs.” They antagonized the function of two of the genome core regulatory sRNAs, GcvB, and FnrS, by mimicking their mRNA substrate sequences to manipulate bacterial pathogenesis (Tree et al., 2014). However, few studies comprehensively investigate the regulatory effects caused by sRNA–sRNA direct interactions.

An advantage of sRNA regulation is its efficiency compared to protein regulators like transcription factors because they do not require translation and act directly on mRNA transcripts (Shimoni et al., 2007). The dynamic nature and low metabolic burden make sRNAs especially suitable to coordinate stress responses including temperature, nutrient, membrane, oxidative, iron, pH, and anaerobic stresses (Gottesman et al., 2006; Hoe et al., 2013; Gottesman, 2019). Ethanol tolerance represents a complex phenotype that sRNAs appear to help regulate. For example, sRNA Nc117 in *Synechocystis* sp. PCC 6803 (Pei et al., 2017) as well as OLE RNA in *Bacillus halodurans* C-125 (Wallace et al., 2012) both appear to protect the cells from ethanol toxicity. However, the mRNA and/or protein targets of these sRNAs are unknown (Nc117) or limited in number (OLE RNA). OLE RNA is known to bind to RNase P as well as a protein (aptly named the OLE-associating protein), which associates to the membrane (Ko and Altman, 2007; Block et al., 2011; Wallace et al., 2012). The lack of network characterization in these contexts has precluded advances in understanding alcohol tolerance and in general sRNA function in non-model organisms. Moreover, as it relates to the specific phenotype of ethanol tolerance, these uncharacterized ethanol-related regulatory RNAs have left unanswered questions of the specific pathways that are co-regulated to naturally grant the ethanol resistance phenotype in some organisms.

*Zymomonas mobilis* is a highly biotechnologically relevant bacterium due to its natural ethanol producing ability up to 12% (v/v) and ethanol tolerance up to 16% (v/v) (Rogers et al., 2007; Franden et al., 2013; Yang et al., 2016a). Over the last 20 years, a variety of *Z. mobilis* strains have been developed through metabolic engineering and directed evolution (Rogers et al., 2007; Yang et al., 2013). The responses of *Z. mobilis* to a variety of stresses, especially ethanol stress, have been explored by transcriptomics and proteomics approaches (Yang et al., 2009, 2013; He et al., 2012a, b; Yi et al., 2015; Zhang et al., 2015). These stress responses are considered a complex phenotype because they trigger the differential expression of large sets of transcripts and proteins with a wide variety of cellular functions. For example, the ethanol stress response has been characterized to include up regulation of protein folding chaperones, DNA repair proteins, and transporters and down regulation of genes related to translation, ribosome biogenesis, and metabolism (He et al., 2012a; Yang et al., 2013; Zhang et al., 2015). These responses are important to the ethanol tolerance in *Z. mobilis* since the ethanol accumulation in cells is toxic, which influences membrane stability, as well as the structure and function of macromolecules such as proteins, nucleic acids, and lipids (Hallsworth et al., 2003). However, regulation mechanisms that cope with these widespread changes remain unclear.

It is likely that these complex phenotypes are made possible by multiple layers of regulation (DNA, RNA, protein) coordinating responses to extracellular environments. Recently, 106 sRNA candidates were identified in *Z. mobilis* by transcriptomics analysis and computationally prediction, where 15 were validated experimentally by Northern blotting analysis and 4 were shown to have differential expression to anaerobic or ethanol stresses (Cho et al., 2014). In this study, we use multiple omics analyses to map the regulatory networks for two of these sRNAs, Zms4 and Zms6, and demonstrate that they have significant impacts on ethanol tolerance and production in *Z. mobilis* through a diverse set of mRNA targets and other sRNA interactions. This work presents the first sRNAs with regulatory binding interactions confirmed in *Z. mobilis* and a large sRNA–sRNA interacting network which has not been widely observed in bacteria.

## Materials and Methods

### Strains and Culture Conditions

The *Z. mobilis* 8b strain, which is an integrant of the ZM4 strain (ATCC 31821), was used in this study. *Z. mobilis* 8b was cultured in RMG media (glucose, 20.0 g/L; yeast extract, 10.0 g/L; KH_2_PO_4_, 2.0 g/L; pH 6.0) at 33°C. *E. coli* DH5α was used for plasmid construction and manipulation, grown in LB media at 37°C. All strains used in this study are listed in Supplementary Table S1.

To generate sRNA overexpression strains, each sRNA was cloned into the *Nco*I-*Sal*I site of the pEZ-tet vector (Yang et al., 2016b) to allow for inducible expression under the P*_*tet*_* promoter. For sRNA co-immunoprecipitation constructs, synthesized 2MS2BD-Zms4/Zm6/control sequences were cloned into pBBR1MCS2-P*_*gap*_* vector (Yang et al., 2014) between *Nhe*I and *Sal*I. All plasmids used in this study are listed in Supplementary Table S1. Strains containing pBBR1MCS2-P*_*gap*_* plasmids were cultured with 350 μg/mL of kanamycin for *Z. mobilis* and with 50 μg/mL for *E. coli*. Overexpression strains containing pEZ-tet vectors were grown with 200 μg/mL spectinomycin for *Z. mobilis* and 50 μg/mL for *E. coli*.

For deletion strain construction, homologous upstream and downstream fragments (each 1 kb) of the target deletion gene were assembled with the spectinomycin gene *aadA*, flanked by LoxP sites, in the middle. Purified PCR product (1 μg) was directly electroporated (200 Ω, 25 μF, 1.6 kV) into *Z. mobilis* competent cells. Electroporated cells were recovered for 6 h and plated onto spectinomycin-containing plates (200 μg/mL). Plated cells were incubated anaerobically in a BD GasPak^TM^ container system for 3–4 days at 33°C. Transformants appearing on the selective plates were cultured and screened for correct size using forward primers of upstream homologous and reverse primer of downstream primer and then sequence-verified. All sequences of primers used in this study are listed in Supplementary Table S2.

Growth rates of *Z. mobilis* strains were evaluated using the Plate Reader (BioTek, Winooski, VT, United States). Biological triplicates of each strain were distributed in triplicates with appropriate antibiotics into 96-well plates with RMG (with and without 6% ethanol) such that each well had a total volume of 200 μL with initial OD_600__nm_ of 0.1. 10 μg/mL anhydrotetracycline was added to each strain to induce sRNA expression from the plasmids. The plate reader measured the turbidity (600 nm) every 0.5 h for 24 h as the cultures grew without shaking at 33°C.

For sRNA induction experiments, each strain was initially grown in biological duplicates in 5 mL RMG culture overnight then transferred into 500 mL with initial OD_600__nm_ of 0.1. Cells were grown anaerobically in sealed flasks containing nitrogen-purged RMG media at 33°C for 4 h to reach OD_600__nm_ around 0.4. Then, 150 mL of cells were collected for transcriptomics and ethanol assay. When OD_600__nm_ reached 0.5 (∼4.5 h of growth), 10 μg/mL anhydrotetracycline was added to each strain to induce sRNA expression from the plasmids. After 0.5 h’s induction (∼5 h of growth), 150 mL of cells were collected to compare the gene expression profile in the middle of exponential phase. In this way, the effect of overexpressing Zms4 and Zms6 on the transcriptome could be confirmed by comparing the samples before and after induction. Final samples were collected during stationary phase (∼12 h of growth). Pelleted cells were stored at −80°C before further processing.

### Ethanol Assay

Ethanol concentrations were measured using the UV-based ethanol assay kit (R-Biopharm, Darmstadt, Germany) according to the manufacturer’s protocol.

### MS2-Affinity Purification of sRNAs *in vivo*

To identify *in vivo* sRNA interactions, each sRNA (Zms4 and Zms6) were tagged with a 2-MS2 binding domain (Said et al., 2009) and isolating by affinity purification with maltose binding protein (MBP) fused to the MS2 coat protein and amylose beads. Briefly, total RNA (100 μL at 500 ng/μL) extracted from *Z. mobilis* strains containing pMS2, pMS2-Zms4, and pMS2-Zms6 was incubated with 10 pmol purified MS2-MBP protein for 1 h at 4°C. Washed amylose beads were incubated with 2MS2BD-Zms4/Zms6/control+MS2-MBP complexes for 2 h at 4°C. Supernatants were removed from the beads by applying a magnet. Beads were washed three times and incubated with 50 μL of elution buffer for 15 min. The elution step was repeated so that 100 μL of each were collected. To precipitate the RNA, equal volumes of isopropanol was added to elution sample and incubated overnight at −20°C. RNA was pelleted at 15,000 rpm for 15 min at 4°C and washed with 1 mL 75% ethanol. The RNA pellets were air-dried then resuspended in 50 μL RNase-free water. RNA samples were stored at −80°C until sequencing.

To find proteins associated with each sRNA *in vivo*, 500 μg of total lysates from *Z. mobilis* strains containing pMS2, pMS2-Zms4, and pMS2-Zms6 were incubated with 10 pmol of purified MS2-MBP protein for 1 h at 4°C. Then, 1 mL Trizol was added to eluted samples for protein purification. After addition of 300 μL of chloroform:isoamyl alcohol mix (v/v 24:1), the samples were inverted for 15 s, and then incubated at 25°C for 3 min. Then, tubes were centrifuged at 13,000 rpm for 10 min. 1.5 mL of isopropanol was then added to the phenol-chloroform layer and mixtures were incubated for 10 min at room temperature and then centrifuged at 12,000 × *g* for 10 min at 4°C to pellet the protein. Pelleted protein was washed with 2 mL of 0.3 M guanidine hydrochloride in 95% ethanol and incubated for 20 min at room temperature then centrifuged at 7,500 × *g* for 5 min at 4°C. Washing steps were repeated twice more. Then, 2 mL of 100% ethanol was added to the protein pellets and samples were centrifuged at 7,500 × *g* for 5 min at 4°C. Air-dried protein pellets were resuspended in 3 × SDS-loading buffer and run 3 mm into the resolving layer of an SDS-PAGE gel (5% stacking; 10% resolving) to concentrate protein before mass spectrometry. The gel was Coomassie stained and total protein bands were excised and stored in destaining solution at 4°C.

### RNA Sequencing and Data Analysis

Total RNA was prepared and purified according to standard methods (DiChiara et al., 2010) for all the growth conditions tested. Prepared RNA was quantified and checked for quality using Bioanalyzer before sequencing. NEBNext^®^ Multiplex RNA Library Prep Set for Illumina^®^ (New England Biolabs Inc.) was used for generating RNA libraries. Sequencing was performed using Illumina^®^ NextSeq 500 with paired-end 2 × 150 bp runs (Genomic Sequencing and Analysis Facility at the University of Texas at Austin).

Adapter sequences and low-quality ends (phred quality < 30) were trimmed from the raw fastq files with cutadapt (v1.3) and reads shorter than 22 nt were discarded (Mortazavi et al., 2008). FastQC was used to verify good read quality for the trimmed files. Reads were aligned to the *Z. mobilis* ZM4 reference genome (taxonomy ID 264203) with BWA-mem (v0.7.12-r1039) (Langmead and Salzberg, 2012). Aligned reads (33∼97 million aligned reads for each sample) were filtered for quality (MAPQ ≥ 10) and sorted by chromosomal coordinates using SAMtools (v0.1.19-44428cd) (Li et al., 2009). The number of reads aligned to each gene was calculated using HTSeq (intersection-non-empty mode for overlaps) (Anders et al., 2015). DESeq2 was used to normalize and identify significantly differentially expressed transcripts between strains (Love et al., 2014). Cytoscape Enrichment Map plugin was used for gene enrichment analysis (Reimand et al., 2019). The experiments for each strain/condition were performed in at least two replicates.

### Mass Spectrometry

Polyacrylamide gel bands containing protein were digested with trypsin. To identify proteins, LC-MS/MS was performed using the Dionex Ultimate 3000 RSLCnano LC coupled to the Thermo Orbitrap Elite with a 2 h run time at the ICMB Proteomics Facility using published methods (Shevchenko et al., 2006). Proteins were searched against the Uniprot *Z. mobilis* ATCC ZM4 database (April 27, 2016) using Sequest HT in Proteome Discoverer 1.4. The identifications were validated with Scaffold v4.4.1 (Proteome Software) with greater than 99.0% probability and with a minimum of two peptides at 99.0% peptide probability. In Scaffold, peptide and protein false discovery rates were both calculated as 0.0%. The experiments for each strain/condition were performed in two replicates.

### Electromobility Shift Assays

Electromobility shift assays (EMSA) were performed to detect the RNA–RNA interactions *in vitro*. Each RNA of interest was amplified from *Z. mobilis* genomic DNA using the primers listed in Supplementary Table S2 and *in vitro* transcribed using MEGAscript T7 Transcription Kit (ThermoFisher). The RNA binding reaction was performed in a 12 μL reaction volume containing 1 × binding reaction buffer [20 mM Tris–HCl (pH 8.0), 1 mM MgCl_2_, 20 mM KCl, 10 mM Na_2_HPO_4_ −NaH_2_PO_4_ (pH 8.0)], 10% glycerol, 5 pmol of ^32^P-labeled sRNA and 10–200 pmol of target RNA. This reaction was denatured at 70°C for 5 min, and then incubated at 37°C for 75 min. Samples were mixed with loading dye (10 mM Tris, 50% glycerol, and 0.0001% wt/vol bromophenol blue) and analyzed by electrophoresis in a 5% non-denaturing polyacrylamide gel (0.5 × TBE, 5% wt/vol acrylamide-bisacrylamide, 5% glycerol, 0.25% ammonium persulfate, and 0.001% TEMED) with 0.5 × TBE running buffer at 4°C for 2.5 h. Next, the gel was placed on a sheet of Whatman grade GB004 blotting paper and dried at 80°C for 60 min (Gel Dryer 583, BioRad). Radioactive bands were visualized using a Typhoon FLA 700 (GE Health Life Science) and analyzed using CLIQS (TotalLab). The fraction bound was then calculated based on the ratio of the intensity of the RNA–RNA complex to the total intensity in each lane, and the dissociation constant (*K*_d_) can calculated as the concentration of the target RNA that showed 50% of binding.

### Detection of RNA-5′UTR Regulation *in vivo*

For 5′UTR sequence determination, the core promoter region and transcriptional start site (TSS) of the target gene were predicted using BPROM. The sequence from the predicted TSS to 99-bp downstream of the ATG was cloned as the 5′ gene fragment to a shuttle vector of dual fluorescence reporting system (Yang et al., 2019). Briefly, using P*_*gap*_* dual report system as backbone, primers of target fragments were designed to contain 15∼20 nucleotides (nts) overlapping regions, which 5′ end overlapped with TSS upstream of P*_*gap*_* and 3′ end overlapped with ATG downstream of GFP. PCR products of target fragments were separated by gel electrophoresis, followed by gel purification, and subsequently quantified using NanoDrop. Fragments and vector were mixed in a molar ratio of 3:1, 0.5 U T5 exonuclease (New England Biolabs Inc.), 0.5 μL buffer 4 (New England Biolabs Inc.), and the final volume was set to 5 μL with ddH_2_O. All reagents were mixed and reacted on the ice for 5 min; *E. coli* chemically competent cells were subsequently added. Cells were plated on LB agar plates with spectinomycin and recombinants were selected by colony PCR and then confirmed by Sanger sequencing (Sangon). The dual-reporter plasmids encoding each target gene was then transformed into the wild type 8b strain and the deletion strains for either Zms4 or Zms6. All sequences of primers used are listed in Supplementary Table S2.

Strains were grown at 30°C for 6–8 h without shaking before washing with phosphate buffered saline (PBS) twice, and then resuspended into PBS to a final concentration of 10^7^ cells/mL. Cells were analyzed by flow cytometry using Beckman CytoFLEX FCM (Beckman Coulter, Inc.) with the PBS as the sheath fluid. The cells fluorescence of Enhanced Green Fluorescent Protein (*EGFP*) were excited with 488 nm and detected with FITC, *mCherry* were excited with 561 nm and detected with PC5.5. Compensation was applied to ensure that the EGFP has minimal affection on the detection of *mCherry* with at least 20,000 events of each sample analyzed. Data were processed via FlowJo software (FlowJo, LLC). The mean fluorescence intensity of triplicates was calculated, then the ratio of ‘average *EGFP*’/‘average *mCherry*’ was used to analyze the interaction of sRNA and 5′-UTR. In addition, the standard deviation was set as error bars. Each experiment was carried out at duplicates.

To detect if sRNA can exert regulations of the targets through the coding regions, an alternative approach was used by replacing the native promoter of the selected targets in the chromosome by a P*_*tet*_* inducible *mCherry* reporter system along with an *aadA* gene as the antibiotics selection marker. The Zms4 and Zms6 overexpression plasmids were then introduced to these strains using the pEZ-tet vector with kanamycin gene replacing the original spectinomycin gene (pEZ-Kana) (Supplementary Table S1). The empty vector was also transformed into these strains as the control. The *mCherry* expression levels of each strain were then measured using the same method as described above.

### Northern Blotting Analysis

Northern blotting analysis was performed by standard methods (Cho et al., 2014). Briefly, DNA oligonucleotide probes specific for each sRNA were labeled using 20 pmol of oligonucleotide in a 20 μL kinase reaction containing 25 μM γ-P^32^ ATP and 20 units T4 polynucleotide kinase (New England Biolabs Inc.) at 37°C for 1 h. Ladder [ΦX174 DNA/*Hin*fI (Promega)] was labeled in the same manner. Total RNA (50–100 μg) from deletion and wild type strains were separated on a 10% denaturing polyacrylamide gel that was then was transferred to a positively charged membrane (Hybond N+, GE Life Sciences) for blotting. Hybridization was performed using Amersham Rapid-hyb buffer (GE Healthcare), following the recommended protocol with overnight incubation at 42°C. After three washes with washing buffer (5 × SSC, 0.1% SDS at 30°C for the first wash and 1 × SSC, 0.1% SDS at 42°C for the second and third washes), membranes were exposed to a phosphor screen overnight. Radioactive bands were visualized using a Typhoon FLA 700 (GE Health Life Science) and analyzed using CLIQS (TotalLab). All probes used in this study are listed in Supplementary Table S2.

## Results

### Zms4 and Zms6 Are Induced by Ethanol and Affect Bacterial Ethanol Tolerance

Four sRNAs (Zms2, Zms4, Zms6, and Zms18) were previously shown as differentially expressed under 5% (v/v) ethanol stress and/or anaerobic conditions (Cho et al., 2014), favoring glucose consumption, enabling higher growth rates and ethanol accumulation (Yang et al., 2009). To determine their direct impacts on the ethanol tolerance phenotype, we overexpressed and deleted each sRNA. However, Zms2 could not be independently overexpressed because it significantly overlaps with its neighboring gene *ZMO1198* and Zms18 could not be fully deleted due to multiple homologous regions in the genome. As such, we focused our study on the characterization of Zms4 and Zms6.

**FIGURE 1:**
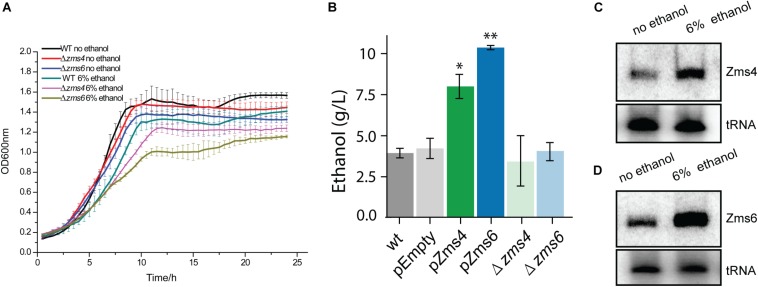
sRNA expression levels affect ethanol tolerance. **(A)** The growth of wild type and Zms4/Zms6 mutant strains with and without 6% (v/v) ethanol supplementation grown anaerobically. Data are presented as means ± SD of biological triplicates. Significant values are ^∗^*p* ≤ 0.05 or ^∗∗^*p* ≤ 0.01 from Student’s *t*-test of each strain compared to wild type. **(B)** Concentration of extracellular ethanol of 150 mL cultures at exponential phase (OD_600__nm_ of 0.4, anaerobically at 33°C). Values represent mean ± SD of biological duplicates. Significant values are ^∗^*P* ≤ 0.05 or ^∗∗^*P* ≤ 0.01 from Student’s *t*-test of each strain compared to pEZ-Empty. **(C,D)** The wild type strain was grown anaerobically at 33°C to reach OD_600__nm_ around 0.5. Ethanol was then added to reach a final concentration of 6%. Cells were collected and total RNA was extracted after 0.5 h. Zms4 **(C)** and Zms6 **(D)** levels were detected by Northern blotting assay using sRNA-specific probes. tRNAs were used as the controls.

After validation of the sRNA deletion and overexpression strains (Supplementary Figure S1), these strains were grown anaerobically in media with and without 6% (v/v) ethanol supplementation to observe the effects of each sRNA on the ethanol survival phenotype. This concentration of 6% (v/v) ethanol was selected to significantly impact cell growth while still maintaining viability (and therefore reproducibility) (Yang et al., 2013). As shown in Figure 1A, Δ*zms4* and Δ*zms6* strains had similar growth to the wild type strain but they showed significantly reduced growth under 6% ethanol stress. Considering the natural ethanol production of *Z. mobilis* and the tolerance effects, we reasoned that the regulatory contribution of Zms4 and Zms6 to the high ethanol tolerance phenotype could also be captured by measuring ethanol production. As shown in Figure 1B, Zms4 and Zms6 overexpression strains showed significantly higher levels of ethanol production relative to strains expressing an empty plasmid and to the wild type strain (with no plasmid). However, the deletion strains remained the same ethanol production ability as the wild type strain. Interestingly, our results also showed natural upregulation of Zms4 and Zms6 when 6% ethanol was added to the media (Figures 1C,D), indicating the possibility that their natural differential expression in *Z. mobilis* plays important roles in increasing fitness to ethanol stress.

### Transcriptomics Analysis Reveal That Gene Networks Affected by Zms4 and Zms6 Are Associated With Ethanol Stress

**FIGURE 2:**
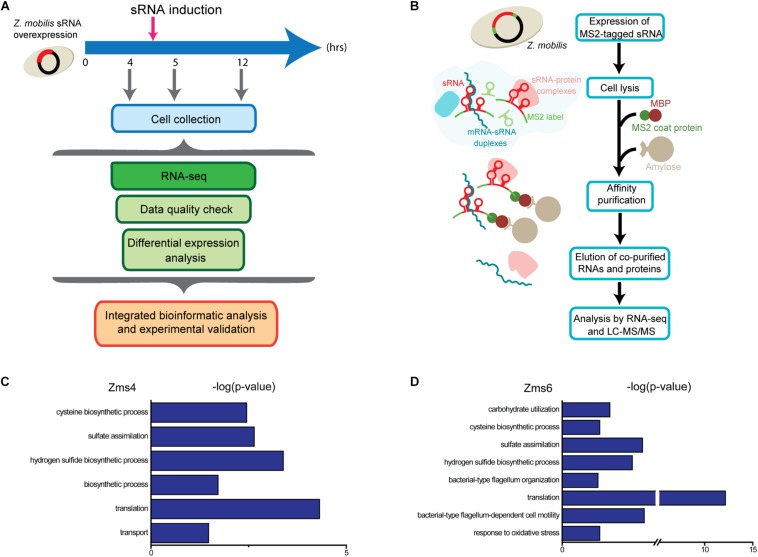
Experimental approaches designed to discern gene networks associated with Zms4 and Zms6. **(A)** Each overexpression strains were grown in 500 mL cultures anaerobically and sRNA plasmid expression was induced at 4.5 h. Portions of cells were collected at 4, 5, and 12 h of growth, representing before induction, after induction, and stationary phase, respectively. RNA was sequenced to discern impacts of Zms4 and Zms6 levels on cellular networks. Differential expression analysis was performed by DESeq2 for RNA-seq data. By integrating the data, patterns of expression at the RNA levels were used to determine most likely targets of sRNA regulation and to infer their regulatory mechanisms. **(B)** *In vivo* RNA interactions were determined by tagging each sRNA with 2MS2 binding domains and isolating them by affinity purification with maltose binding protein (MBP) and amylose beads. Upon elution, physically associated transcripts and proteins were identified by RNA-seq and mass spectrometry. **(C)** Gene ontology (GO) analysis of enriched pathways upon Zms4 induction relative to all detected genes. **(D)** GO analysis of enriched pathways upon Zms6 induction relative to all detected genes.

As global regulators, sRNAs can interact with multiple mRNA and protein targets to coordinate complex phenotypes. Given that sRNAs can repress or activate gene expression, we expect that altering their stoichiometry will lead to significant changes on the innate levels of their direct or indirect targets. To uncover the networks of targets affected by Zms4 and Zms6, we used an Integrative FourD-Omics (INFO) approach (Sowa et al., 2017), useful for identification of regulated targets and regulatory mechanisms underlying sRNA-driven systems. This integrated analysis (Figures 2A,B) included collection of transcriptome and MS2-affinity purification coupled with RNA sequencing (MAPS) profiles evaluated in Zms4 and Zms6 overexpression strains.

For these analyses, cells were collected before induction during the exponential-phase (4 h), 0.5-hour post-induction (5 h), and 7 h post-induction in the stationary phase (12 h) (Figure 2A). From these samples, RNA was extracted by standard methods and further characterized by RNA-seq in duplicates. 1939 genes and sRNAs (91.77% of 2113 uncovered genes/sRNAs) were detected using DESeq2 and 723 genes and sRNAs (34.22%) were observed to be differentially expressed by at least a twofold increase or decrease upon Zms4 and Zms6 induction. From this analysis, 504 genes and 42 sRNAs were significantly affected in a Zms4-dependent manner (*p*_*adj*_ < 0.05); and 418 genes and 35 sRNAs were significantly affected in a Zms6-dependent manner (*p*_*adj*_ < 0.05) (Supplementary Table S3). The induction of Zms4 especially affected expression of transcripts involved in transport and biosynthetic processes (Figure 2C) and the induction of Zms6 especially influenced oxidative stress response, cell motility/flagellum organization, and carbohydrate utilization (Figure 2D). Interestingly, more than half of them (249 genes and 27 sRNAs) were influenced by both Zms4 and Zms6, such as translation, hydrogen sulfide biosynthetic processes, sulfate assimilation, and cysteine biosynthetic processes (Supplementary Table S3 and Figures 2C,D). Several transcripts and proteins potentially regulated by Zms4 and Zms6 have been previously reported to be differentially expressed under ethanol stress (Table 1). Given that Zms4 and Zms6 are naturally up-regulated upon ethanol stress, these observations evidence that many transcripts and proteins associated with cellular levels of Zms4 and Zms6 are tightly linked to ethanol tolerance pathways. In addition, several genes directly involved in ethanol metabolism were also found differentially expressed. For instance, alcohol dehydrogenase 1 (*adhA*), which facilitates the interconversion between alcohols and aldehydes, was significantly upregulated by Zms4 and Zms6 overexpression. The expression of *hfq* gene and its 5′-UTR were also significantly upregulated upon the induction of both Zms4 and Zms6, suggesting that Zms4 and Zms6 may affect these important pathways by modulating the expression of other Hfq-dependent sRNAs (although Hfq has not been yet demonstrated to be functional as an sRNA chaperone in *Z. mobilis*).

**TABLE 1:** List of transcripts and proteins differentially regulated by both ethanol stress and sRNA overexpression in *Z. mobilis*.

**Transcripts differentially expressed under both 5% ethanol stress (He et al., 2012a) and Zms4 over expression**	
ZMO0265	Aspartyl protease
ZMO0374	Levansucrase (beta-D-fructofuranosyl transferase) (sucrose 6-fructosyl transferase)
ZMO0447	Uncharacterized protein
ZMO0614	Flagellar basal body rod protein FlgB
ZMO0924	Protein translocase subunit SecA
ZMO1045	Phosphate-selective porin O and P
ZMO1055	Diguanylate cyclase/phosphodiesterase
ZMO1065	Phage shock protein C, PspC
ZMO1262	Binding-protein-dependent transport systems inner membrane component
ZMO1285	Glucose-methanol-choline oxidoreductase
ZMO1426	DNA repair protein RadC
ZMO1458	Uncharacterized protein
ZMO1522	TonB-dependent receptor
ZMO1647	Transcription-repair-coupling factor (TRCF)
ZMO1649	Uracil-DNA glycosylase superfamily
ZMO1696	Zinc-binding alcohol dehydrogenase family protein
ZMO1802	Integration host factor subunit beta
ZMO1851	Uncharacterized protein
ZMO1855	Transcriptional regulator, GntR family with aminotransferase domain
ZMO1882	Uncharacterized protein
ZMO1961	Uncharacterized protein
ZMO2030	50S ribosomal protein L32
**Transcripts differentially expressed under both 5% ethanol stress (He et al., 2012a) and Zms6 over expression**	
ZMO0265	Aspartyl protease
ZMO0374	Levansucrase (Beta-D-fructofuranosyl transferase) (Sucrose 6-fructosyl transferase)
ZMO0899	NAD+ synthetase
ZMO0917	2-nitropropane dioxygenase, NPD
ZMO0952	tRNA (cytidine(34)-2′-*O*)-methyltransferase (tRNA (cytidine/uridine-2′-*O*-)-methyltransferase TrmL)
ZMO0998	Peptide methionine sulfoxide reductase MsrA (Protein-methionine-*S*-oxide reductase)
ZMO1012	Uncharacterized protein
ZMO1030	Uncharacterized protein
ZMO1067	Fe-S metabolism associated SufE
ZMO1295	7-carboxy-7-deazaguanine synthase (CDG synthase) (queuosine biosynthesis protein QueE)
ZMO1311	LPS-assembly protein LptD
ZMO1458	Uncharacterized protein
ZMO1473	Uncharacterized protein
ZMO1522	TonB-dependent receptor
ZMO1649	Uracil-DNA glycosylase superfamily
ZMO1697	Zinc-binding alcohol dehydrogenase family protein
ZMO1855	Transcriptional regulator, GntR family with aminotransferase domain
ZMO2034	Conserved hypothetical replication initiator and transcription repressor protein

### Identification of Potential Direct Targets of Zms4 and Zms6

To identify specific transcripts and proteins that *directly* interact *in vivo* with Zms4 and Zms6, we conducted a genome-wide MAPS approach (Figure 2B) (Lalaouna and Masse, 2015). For these experiments, each sRNA was 5′ end tagged with a 2-MS2-binding domain (sequences in Supplementary Table S2). The tagged sRNAs were expressed in the wild type strain using the pBBR1MCS2-Pgap vector (Yang et al., 2014). Bound complexes were collected at the exponential phase (OD_600__nm_ of 0.6) and isolated using maltose binding protein (MBP) columns. Transcripts and proteins co-precipitated with each sRNA were identified by RNA-sequencing and Mass spectrometry. Transcripts and proteins with at least 1.5-fold enrichment in the pMS2-Zms4 and pMS2-Zms6 strains relative to the pMS2 control (lacking sRNA expression) were analyzed further as potential sRNA targets (Supplementary Tables S4, S5).

**FIGURE 3:**
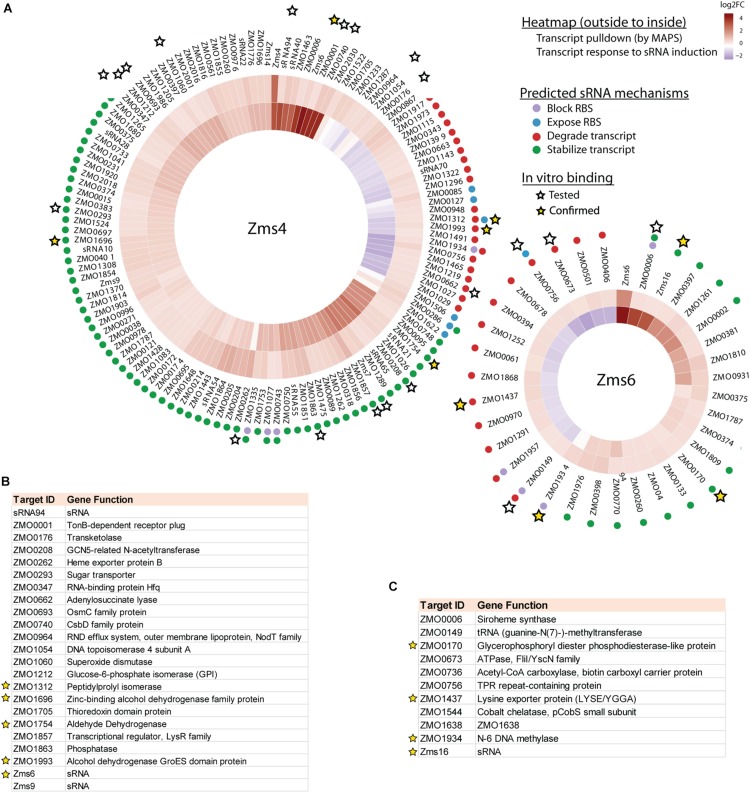
Integrated omics analyses reveal most likely targets of Zms4 and Zms6 regulation. **(A)** Heat map shows the genes that co-immunoprecipitated with Zms4 and Zms6 and also showed patterns of gene expression consistent with canonical sRNA mechanisms: blocking or exposing the RBS and degrading or stabilizing the transcript (colored dots). Heat map tiles (outside to inside) show the log_2_ fold changes of the sRNA-immunoprecipitation enrichment compared to the control, and the transcript expression level at 5 h (after sRNA induction) vs. 4 h (before induction). Multiple potential targets with a variety of predicted mechanisms were tested for direct binding with each sRNA *in vitro* (stars) and several were confirmed (yellow stars). **(B)** The list of selected Zms4 targets tested by EMSA. The confirmed targets are marked by yellow stars. **(C)** The list of selected Zms6 targets tested by EMSA. The confirmed targets are marked by yellow stars.

123 and 35 transcripts were pulled down by MS2-MBP-Zms4 and MS2-MBP-Zms6, respectively (Figure 3A and Supplementary Table S4). Based on the observed expression patterns in the transcriptomics data, potential mechanisms of regulation were predicted (Figure 3A). Zms4 was hypothesized to directly repress 33 transcripts by inducing transcript degradation or blocking the ribosome binding site (RBS) sequence and to directly activate 88 genes by transcript stabilization or exposure of the RBS. Similarly, Zms6 was hypothesized to directly up-regulate 16 transcripts and directly down-regulate 20 transcripts by the same corresponding mechanisms. Functionally, these potential targets are involved in differently pathways, including transport, DNA repair, cell motility/flagellum organization and oxidative-reductase stress response. One of our most interesting observations was that the Zms4 and Zms6 pulldowns were enriched for several other sRNAs that were previously identified to naturally respond to ethanol stress in *Z. mobilis* (Cho et al., 2014), which is consistent with our transcriptomic data showing altered expression of several previously identified sRNAs (Cho et al., 2014) by Zms4 and Zms6 induction (Supplementary Table S3). Importantly, Zms6 was also significantly enriched in the Zms4 pulldown, and 26 transcripts were enriched in both Zms4 and Zms6 (Supplementary Table S4). These observations strengthened the possibility that Zms4 and Zms6 interact with each other and subsequently seed a multi-sRNA network. Another observation was the potential that Zms4 and Zms6 have both activation and repression regulatory capabilities, an interesting possibility given the rarer activation role that has been assigned to sRNAs from studies in model bacteria.

To analyze the potential functional dependence of Zms4 and Zms6 on cellular factors (i.e., chaperones, etc.), we also analyzed proteins that were uniquely enriched by co-precipitation with Zms4 and Zms6 relative to the pMS2 control (lacking sRNA expression) (*p* < 0.05, Students’ *T*-test, Supplementary Table S5). Given that our major focus was to identify mRNA targets, we were most interested in proteins that co-immunoprecipitated with both sRNAs as potential indication of specific cellular co-factors that could play important roles in the biology of sRNAs in *Z. mobilis*. This was especially interesting given the lack of sRNA characterization that has been done in this non-model organism. Given that the transcription termination/antitermination protein (NusA, ZMOb_1083) was co-immunoprecipitated with both sRNAs, it is likely that NusA-dependent transcription processing is relevant to these sRNAs in *Z. mobilis*. The biological significance (if any) of the second protein enriched in both Zms4 and Zms6 [3-isopropylmalate dehydratase large subunit (LeuC, ZMOb_0723)] is less clear as no RNA interactions are predicted for this protein and no homologs of this protein that have been shown to be regulated by sRNAs in other species. It is also worth noting that the Hfq protein, was not detected to co-precipitate with these sRNAs, indicating the possibility that it is not essential for Zms4 and Zms6 function or that it might not serve as a strong general sRNA–mRNA chaperone in this organism. Although we cannot exclude the possibility that some proteins attached *in vivo* were lost in our purification procedure or difficult to detect by LC-MS/MS (Kaboord et al., 2015), the lack of Hfq binding to these particular sRNAs was also observed *in vitro* (Supplementary Figure S2A). The expression of Zms4 and Zms6 was also not affected by the deletion of *hfq* (Supplementary Figure S2B), although we showed here that Hfq is essential for the survival of *Z. mobilis* under both normal condition and 6% ethanol stress (Supplementary Figure S2C). However, our transcriptomics data showed that the expression of *hfq* gene and its 5′UTR was upregulated significantly upon Zms4 and Zms6 induction (Supplementary Table S3). These data suggest that Zms4 and Zms6 could serve as advanced regulators over other sRNAs through affecting the expression of *hfq*. Overall, our characterization of Hfq interactions indicate that these two sRNAs function in an Hfq-independent manner but potentially affect a wide range of sRNAs through modulating *hfq* expression indirectly. It is worth noting that no homology of other known sRNA chaperons, like ProQ, has been found in *Z. mobilis*.

### Confirmation of Multiple Direct RNA Targets

Given the non-specific interactions that can be captured via pull-down approaches like MAPS (which are likely amplified by the sensitivity of RNA-seq approaches), we next validated physical interactions of the predicted mRNA targets and the corresponding sRNA. We selected 23 target candidates of Zms4 and 11 target candidates of Zms6 for *in vitro* testing (marked with a star in Figure 3A). We selected our candidates based on their potential relevance to ethanol tolerance as judged by our transcriptomics data and previous omics studies in *Z. mobilis* (Yang et al., 2009; Yi et al., 2015) and by whether they are independently transcribed (whether their 5′-UTRs significantly overlap with neighboring gene coding regions). For these assays, the 5′ region (∼−200 to +100 nt relevant to the start codon) of each mRNA target was initially screened for binding with the full sRNA transcript [as previously characterized by RACE analysis (Cho et al., 2014)] using EMSAs with 5 pmol sRNA and excessive targets (100 pmol). We selected these regions given that most sRNAs are found to base pair within the 5′-UTR region of the target they regulate (Gottesman and Storz, 2011). We confirmed interactions for 5 of the 23 RNAs tested as potential Zms4 targets and for 4 of the 11 RNAs tested as potential Zms6 targets by EMSA analysis (Figures 3B,C, Table 2, and Supplementary Figure S3). We confirmed two alcohol dehydrogenase genes (*ZMO1696* and *ZMO1993*), a peptidylprolyl isomerase gene (*ZMO1312*) and an aldehyde dehydrogenase gene (*ZMO1934*) as Zms4 targets, suggesting that both the ethanol synthesis and anabolism pathways are potentially regulated by this sRNA directly. As for Zms6, the confirmed targets include an N-6 DNA methylase gene (*ZMO1934*), a lysine exporter encoding gene (*ZMO1437*) and a glycerophosphoryl diester phosphodiesterase encoding gene (*ZMO0170*), which indicates Zms6 might affect the ethanol related pathways in an indirect manner through these genes. Notably, we also confirmed the binding between Zms4 and Zms6 as well as the interaction between Zms6 and another previously identified sRNA, Zms16 (Cho et al., 2014). This supports our hypothesis that these sRNAs interplay with each other and form a multi-sRNA network.

**TABLE 2:** Confirmed targets of Zms4 and Zms6 regulation.

**sRNA**	**Targets**	**Description**	**Predicted binding sites**	***K*_d_ (pmol)**
Zms4	*ZMO1696*	Zinc-binding alcohol dehydrogenase family protein	−11 ……−1	46.8
	*ZMO1754*	Aldehyde dehydrogenase	−28 ……−17	117.9
	*ZMO1993*	Alcohol dehydrogenase GroES domain protein	−51 ……−38	107.3
	*ZMO1312*	Peptidylprolyl isomerase	+40 ……+53	600.0
	Zms6	sRNA	99 ……117	42.8
Zms6	*ZMO1934*	N-6 DNA methylase	−38 ……+26	20.9
	*ZMO0170*	Glycerophosphoryl diester phosphodiesterase-like protein	−3 ……+12	53.2
	*ZMO1437*	Lysine exporter protein	+28……+75	132.0
	Zms16	sRNA	303 ……318	27.7

**FIGURE 4:**
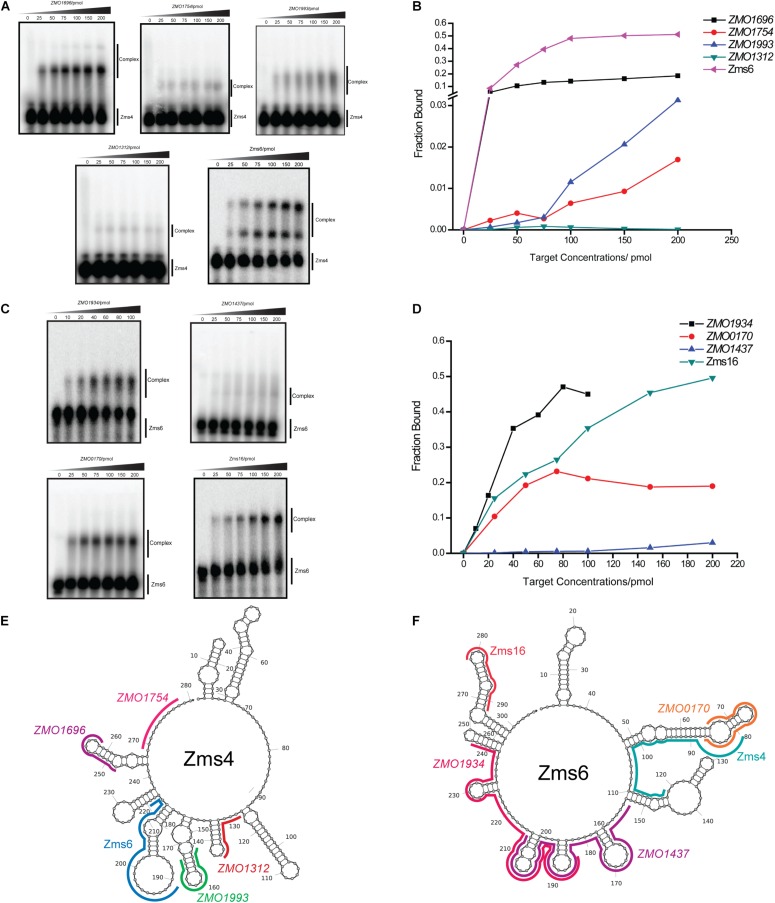
sRNA-target interacting pairs verified by EMSA. The internally labeled Zms4/Zms6 (5 pmol) was incubated with increasing concentrations (0–200 pmol) of target RNAs. *ZMO1696*, *ZMO1754*, *ZMO1993*, *ZMO1312*, and Zms6 were confirmed to bind to Zms4 *in vitro* **(A)** with various affinities **(B)**. In the case of Zms6, two complexes were detected. *ZMO1934*, *ZMO1437*, *ZMO0170*, and Zms16 were confirmed to bind to Zms6 *in vitro* **(C)** with various affinities **(D)**. Binding site locations of the confirmed targets of Zms4 and Zms6 were predicted by IntaRNA and mapped onto the secondary structure of sRNAs inferred by NUPACK (**E,F**).

To better quantify how strong these interactions are and to exclude the possibility that the observed interactions did not result artificially from excessive usage of RNA targets, we incubated 5 pmol of sRNA with increasing concentrations (0∼200 pmol) of various targets and calculated their dissociation constant (Figures 4A–D and Table 2). Our results demonstrated that Zms4 formed stable complexes with high affinity (46.8 pmol and 42.8 pmol) with *ZMO1696* and Zms6. Interestingly, for these two targets, two distinct complexes were observed, indicating a potential 2:1 binding stoichiometry between Zms4 and these mRNAs. In contrast, the affinities of RNA complexes between Zms4 and *ZMO1993*, *ZMO1754* and *ZMO1312* were weaker (107.3 pmol, 117.9 pmol, and 600 pmol). For Zms6, *ZMO1934* and Zms16 showed the strongest affinity (20.9 pmol and 27.7 pmol), while the binding of other complexes (*ZMO1437* and *ZMO0170* mRNAs) was significantly weaker (132 pmol and 53.2 pmol).

We subsequently predicted the binding sites of these confirmed targets using IntaRNA (Supplementary Figure S4). The results showed that the binding sites on the mRNA targets localize in traditional 5′UTR regions or near the start codon in the coding region. For Zms4 targets, the binding sites with *ZMO1696*, *ZMO1993*, and *ZMO1754* were predicted to be located in 5′-UTR region, and the binding site with *ZMO1312* was predicted to be located in the coding region. In terms of Zms6 targets, the binding site for *ZMO1437* was predicted to be located in the coding region, while the binding sites for *ZMO0170* and *ZMO1934* were predicted to be located in both the 5′-UTR and the coding region. The predicted binding locations of these targets were then mapped to each sRNA’s secondary structure inferred from NUPACK (Zadeh et al., 2011). As shown in Figures 4E,F, Zms4 and Zms6 contain multiple functional sites that potentially contribute to multi-tasking in their function. Moreover, these sites are predicted to occupy regions of high and low hybridization efficacy, as identified by the InTherAcc biophysical model (Vazquez-Anderson et al., 2017), which could explain the observed differences in binding affinities. These results also imply that Zms4 and Zms6 could bind and regulate multiple targets simultaneously and efficiently.

### Confirmation of the Predicted Binding Sites for Important Targets by Mutagenesis Analysis

To confirm computationally predicted binding sites, we selected some of the important RNA targets that are either confirmed to be ethanol-relevant or displaying strong affinity, and experimentally confirmed the actual binding site locations of them by EMSAs. For Zms4, we studied *ZMO1696*, *ZMO1993* and Zms6 because they exhibited strong affinities. *ZMO1754* was also selected since this gene encodes aldehyde dehydrogenase that coverts acetaldehyde, a compound generated from ethanol anabolism, to acetate, and thus directly related to ethanol tolerance. For Zms6, we studied *ZMO1934* and Zms16 considering they showed higher affinity than the other two targets and are likely the most important targets of Zms6.

**FIGURE 5:**
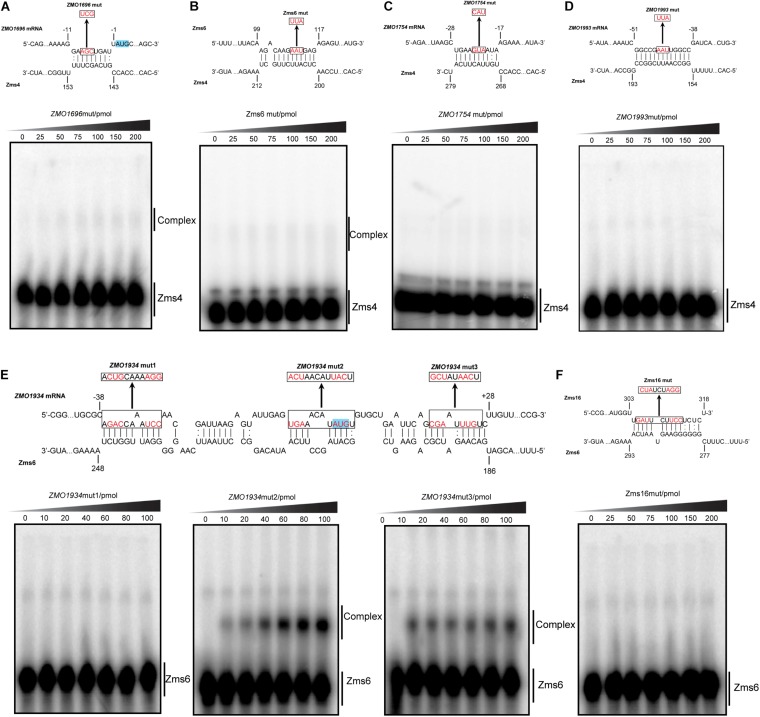
Confirmation of the predicted binding sites for important targets by mutagenesis following EMSA assays. *ZMO1696*, *ZMO1754*, *ZMO1993*, and Zms6 as Zms4 targets **(A–D)** and *ZMO1934* and Zms16 as Zms6 targets **(E,F)** were selected. The mutation introduced in each binding sites are shown by red and the translation start codons are highlighted in blue. The target variants carrying basepairing substitutions were then tested for their binding ability with Zms4/Zms6 under the exactly same conditions as we tested the wild-type target RNAs.

As exemplified in Figure 5, we mutated several nucleotides in the predicted base pairing regions for each target RNA and the ability of mutated targets to form complexes with Zms4 and Zms6 were then tested. The data showed that the complexes of Zms4–*ZMO1696*mut and Zms4–Zms6mut almost disappeared (Figures 5A,B), while complex formations of *ZMO1754*mut and *ZMO1993*mut with Zms4 were completely abolished (Figures 5C,D). These results are consistent with the IntaRNA predictions that these mRNA targets interact with Zms4 though the 5′-UTR regions (Supplementary Figure S4). Through similar testing, the binding sites on *ZMO1934* and Zms16 essential for their interactions with Zms6 were also confirmed (Figures 5E,F). Specifically, Zms6 was predicted to interact with the *ZMO1934* mRNA through extensive base pairing (over 50 nucleotides), which overlaps with both 5′-UTR and coding regions. We then created three mutations for this target and found that only *ZMO1934*mut1 failed to interact with Zms6, while the other two are still able to form stable complexes with this sRNA (Figure 5E). However, quantification of the data showed that *ZMO1934*mut2 and *ZMO1934*mut3 bind to Zms6 with much lower affinities (39.4 pmol and 44.1 pmol, Supplementary Figure S5), which indicates cooperation between all of the three sites and the region from −38 to −27 nt to be the dominating site. Taken together, these results correlate well with the prediction of the basepairing interactions and suggest that these mRNA targets are potentially regulated by Zms4 or Zms6 through 5′-UTR regions or near the start codon in the coding region.

### Detection of sRNA-Target Regulation and sRNA–sRNA Crosstalk *in vivo*

Then we used fluorescent reporter assays to confirm that the sRNAs can exert a regulatory effect on their mRNA targets *in vivo* through the predicted binding sites. In this assay, the 5′ UTRs of the RNA targets shown to interact with Zms4 (*ZMO1993*, *ZMO1312*, *ZMO1696*, and *ZMO1754*) or Zms6 (*ZMO1934*, *ZMO0170*, and *ZMO1437*) *in vitro* were cloned upstream of *EGFP* and expressed under the constitutive strong promoter *P*_*gap*_ in a dual-reporter-gene system (Yang et al., 2019) (Supplementary Figure S6A). The vector also contains *mCherry* under the *P_*lacUV*__5_* promoter as a control for the expression level so the specific effect of the sRNA on each gene’s 5′-UTR can be observed. The vectors containing the 5′-UTR of targets were transformed into the wild type 8b strain and Zms4/Zms6 deletion strains. Unfortunately, we were only able to test the 5′-UTR of *ZMO1993* and *ZMO1754* for Zms4 and the 5′-UTR of *ZMO1934* and *ZMO0170* for Zms6 as no fluorescence of *ZMO1696*, *ZMO1312* (for Zms4), and *ZMO1437* (for Zms6) could be detected (implying that the reporter system did not work for these constructs).

**FIGURE 6:**
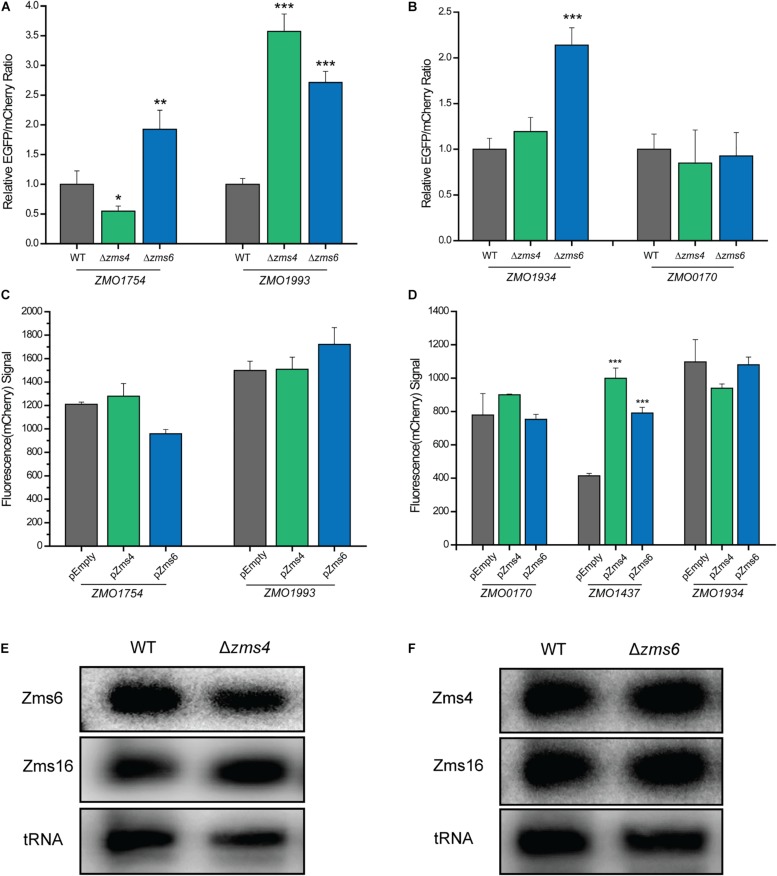
sRNA-target regulation *in vivo*. The regulation of Zms4 and Zms6 on the 5′-UTR expressions for the selected targets [Zms4 targets shown in **(A)** and Zms6 targets shown in **(B)**] were determined using the Dual reporter system. The Zms4 and Zms6 deletions strains carrying the Dual-reporter systems for each target tested were grown 6–8 h and then fluorescence was measured by flow cytometry in biological triplicates. Mean fluorescence values were normalized by the wild type level of *EGFP*/*mCherry* signal and error bars represent standard deviation (^∗^*P* ≤ 0.05, ^∗∗^*P* ≤ 0.01 or ^∗∗^*P* ≤ 0.001 from Student’s *t*-test of each strain compared to wild type). The regulation of Zms4 and Zms6 on the coding region expressions for the selected targets [Zms4 targets shown in **(C)** and Zms6 targets shown in **(D)**] were determined using the promoter replacement system. Zms4 and Zms6 overexpression strains constructed using the strains that contain the replaced promoter for each target were grown 24 h and then fluorescence was measured by flow cytometry in biological triplicates. The strains harboring the empty vector were used as the control strains. Mean fluorescence values were calculated and error bars represent standard deviation (^∗∗^*P* ≤ 0.001 from Student’s *t*-test of each strain compared to the control strain). The regulatory outcomes of *in vivo* sRNA interactions were determined using Northern blotting analysis. Total RNAs were extracted from wild type and Zms4/Zms6 mutant strains **(E,F)**. Zms4, Zms6, and Zms16 levels were then detected sRNA-specific probes. tRNAs were used as the controls.

The relative *EGFP* and *mCherry* expression levels of each strain were then quantified using a flow cytometer. The 5′-UTR of *ZMO1993* exhibited a higher relative *EGFP*/*mCherry* fluorescence ratio in Zms4 deletion strain than that of the wild type strain, indicating the potential negative regulatory effect of Zms4 on *ZMO1993* (Figure 6A). For *ZMO1754*, the deletion of Zms4 leads to a significant decrease in *EGFP*/*mCherry* fluorescence ratio relative to the wild type strain (Figure 6A), which suggests that Zms4 positively regulates *ZMO1754* through interactions with its 5′-UTR. These results are consistent with our hypothesized mechanism by IntaRNA prediction and transcriptomic data that Zms4 protects *ZMO1754* transcript from degradation while promotes degradation of *ZMO1993* transcript by binding to the 5′-UTR regions of these mRNAs (Figure 3A and Supplementary Figure S4A). Zms6 appears to negatively regulate *ZMO1934 in vivo* through its 5′-UTR, since the *EGFP*/*mCherry* fluorescence ratio increased in the Zms6 deletion strain relative to the wild type strain (Figure 6B). However, no significant effect of Zms6 on the *ZMO0170* 5′-UTR was observed (Figure 6B). This is likely due to the predicted competitive binding of Zms4 with a higher affinity *in vivo* on the same region where Zms6-*ZMO0170* base-pairing locates (Figure 4F).

Since the binding sites on some of the targets also locate in the coding regions (i.e., *ZMO1934* and *ZMO1437*, Supplementary Figure S4B), we used a promoter replacement system (Supplementary Figure S6B) to observe the *in vivo* regulation of Zms4 and Zms6 on the coding regions for some of the important targets. The native promoter regions of the confirmed targets (*ZMO1993* and *ZMO1754* for Zms4, and *ZMO1934* and *ZMO0170* for Zms6 were replaced by a P*_*tet*_* induced *mCherry* reporter system along with an *aadA* gene. We also selected *ZMO1437* because this gene (lysine exporter) was previously reported to improve the tolerance to phenolic aldehydes (Yi et al., 2015) and thus may also play important roles in the Zms6 regulation. The fluorescence signals of *mCherry* were then measured in Zms4 and Zms6 overexpression strains as compared to the signals from the wild type strain carrying the empty vector (the control strain). The signals of *ZMO1993* and *ZMO1754* in the Zms4 overexpression strain were comparable to the control strain (Figure 6C), and the signals from *ZMO1934* and *ZMO0170* upon Zms6 overexpression were also similar to the signals from the control strains (Figure 6D). These are consistent of our predictions and mutagenesis experiments that the binding sites of these targets are in the 5′UTR region instead of the coding regions. On the contrary, the *mCherry* signal of *ZMO1437* in the Zms6 overexpression strain were significantly greater than the control strain (Figure 6D). From this data, we can infer that Zms6 likely activates the expression of *ZMO1437* through the interaction with the coding region, as consistent with our IntaRNA prediction (Supplementary Figure S4B). These experiments confirm that binding of Zms4 and Zms6 to their confirmed mRNA targets can exert regulatory effects *in vivo*.

Given the lack of overlap between the confirmed set of direct mRNA targets of Zms4 and Zms6 and the fact that these two sRNAs share some important pathways associated with alcohol tolerance (Figures 2C,D and Supplementary Table S3), we hypothesized that Zms4 and Zms6 could mediate crosstalk between these targets. In this way, these two sRNAs can link regulation of a broader set of pathways, establishing a wider regulatory network of relevance to a complex phenotype like ethanol tolerance. Thus, we further tested if one sRNA can affect the confirmed targets of the other one. Using the dual reporter system, we observed that the relative *EGFP/mcherry* fluorescence ratios of *ZMO1754* and *ZMO1993* 5′-UTRs were significantly higher in Zms6 deletion strain compared to the wild type strain (Figure 6A). In contrast, the relative *EGFP/mcherry* fluorescence ratios of *ZMO1934* and *ZMO0170* 5′-UTRs were comparable than those in the Zms4 deletion strain in comparison with the wild type strain (Figure 6B). However, using the promoter replacement system, we found that Zms4 overexpression strain also exhibited a significantly higher fluorescence signal than the control strain (Figure 6D), while the coding regions of other targets (*ZMO1754*, *ZMO1993*, *ZMO1934*, and *ZMO0170*) were not influenced by Zms4 or Zms6 (Figures 6C,D). These results showed that the interaction between Zms4 and Zms6 could result in the crosstalk of the regulations on some of their direct targets. More importantly, we found that deletion of Zms4 also did not affect transcript levels of Zms6 and vice versa (Figures 6D,E). Similarly, Zms16 levels also didn’t shown any obvious change in Zms4 and Zms6 deletion strains (Figures 6D,E). This indicates that, unlike the mRNA targets, the sRNA–sRNA interplays (Zms4–Zms6 or Zms6–Zms16 interaction) are probably through competition binding or structural changes instead of changing the expressions.

### Combinatorial Effects of sRNAs on Ethanol Tolerance Show Complex Effect

Considering the interactive network of multiple sRNAs potentially seeded by Zms4 and Zms6 and some pathways specifically mediated by Zms4 or Zms6 (i.e., transport process regulated by Zms4 and hydrogen sulfate biosynthetic process regulated by Zms6), we further investigated the combinatorial effect of sRNAs on ethanol tolerance. For these experiments, strains overexpressing each possible combination pairs of Zms4, Zms6, and Zms16 were developed, as well as a strain expressing all three. As Supplementary Figure S7 shows, most of the combination strains exhibited growth rates with significantly enhanced growth under ethanol than the wild type strain with empty plasmid in 6% (v/v) ethanol. The strain combination of overexpression of all three (pZms4-6-16 strain) shows the highest growth rate of all the strains tested under ethanol stress, while its growth rate is comparable to the empty vector control under no ethanol condition. These results confirmed the combinatorial importance of these sRNAs to growth on the ethanol stress, and the possibility that sRNAs could work in synergy to co-regulate the ethanol tolerance and confirmed that the interactions between Zms4 and Zms6 could lead to the cross-talk regulations on their targets *in vivo*.

## Discussion

The survival of bacteria is highly dependent on their ability to sense and adapt to changes in the environment, which entails a coordinated regulation of large networks of gene/protein expression (Hoe et al., 2013). In this study, two sRNAs, Zms4 and Zms6, that are naturally differentially expressed under ethanol stress in *Z. mobilis* are shown to be key to ethanol tolerance and shown to coordinate a large network of gene regulation that includes sRNA–sRNA interactions. Without these sRNAs, cells are highly sensitive to ethanol stress, and that by manipulating their cellular levels, ethanol tolerance can be improved. To our knowledge, this represents the first large sRNA–sRNA interacting network in bacteria.

In this study, multi-omics analyses play a key role in uncovering the network of sRNAs and their important roles in ethanol stress response (Figures 2A,B and Supplementary Table S3). Our transcriptomics experiment identified 34.22% of *Z. mobilis* genes involved in a wide range of cellular processes that are differentially regulated by the overexpression of Zms4 and Zms6 (Supplementary Table S3). This global level of transcriptional change is not surprising because both Zms4 and Zms6 greatly affect the ethanol tolerance, a complex phenotype (Figure 1A), and regulate several global transcriptional regulators or two-component systems. Many of the changed transcripts are associated with translation, hydrogen sulfide biosynthetic process, sulfate assimilation, and cysteine biosynthetic processes. These pathways represent many of the same basal metabolic function that are negatively impacted by ethanol stress and have therefore been implicated in ethanol toxicity (Yang et al., 2009, 2013; He et al., 2012a, b; Yi et al., 2015; Zhang et al., 2015). Previous studies also showed that accumulation of ethanol inside the cells can also promote changes in membrane composition and affect membrane-related processes such as energy generation and transport (Hallsworth et al., 2003). Correspondingly, we found that a large number of transporters, electron transfer genes, DNA repair genes and membrane associated genes are affected by Zms4 or Zms6, either positively or negatively, which may be important to combat the negative consequences caused by the ethanol accumulation.

Although several potential mRNA targets were tested, EMSA assays revealed a few mRNAs to form stable complexes with Zms4 and Zms6 (Figures 4A–D). The targets not detected to bind might be highly dependent on the *in vivo* environment and/or bind to other locations of the mRNAs, outside the 5′ regions tested in this study. Alternatively, some RNA-binding protein may exist to simulate the formation of multiple RNA–RNA complexes *in vivo* which we didn’t include in the EMSA assays. RNA–RNA binding site interactions can be difficult to predict computationally due to the sensitivity of these interactions to structural complexity and *in vivo* variables beyond current modeling capabilities. Interestingly, most of the Zms4 and Zms6 targets occupy different sites on the sRNAs (Figures 4E,F). This observation indicates that the sRNAs could potentially co-regulate multiple targets simultaneously, suggesting flexibility and efficiency of the Zms4 and Zms6 regulation.

Although the inferred mechanisms of Zms4 and Zms6 regulation on all the targets were not specifically validated in this study, the hypothesized mechanisms are consistent with those observed in previous studies about *Z. mobilis* and other organisms. Using *in vivo* reporter systems, we uncovered that Zms4 positively regulates aldehyde dehydrogenase gen *ZMO1754* and represses the expression of alcohol dehydrogenase gene *ZMO1993* through interaction with their 5′ UTR regions (Figure 6A). Aldehyde dehydrogenase (ZMO1754/SsdA) is responsible for the oxidation of acetaldehyde to acetate and is expressed eightfold higher in xylose-only media compared with glucose-only media (Mohagheghi et al., 2014). In the metabolism, acetaldehyde represents a branching point: converted to either ethanol by AdhA/B or acetate by SsdA (ZMO1754) (Zhang et al., 1995; Edenberg, 2007; Yi et al., 2015). Interestingly, the elevated *adhA* levels were also found by the overexpression of Zms4 in our transcriptomic data. Considering that any of these three compounds cause stress to the cell, one important role of Zms4 could be to shift flux away from ethanol production by stabilizing this particular transcript during stress, and/or to reduce acetaldehyde/ethanol accumulation. *ZMO1993* encodes an alcohol dehydrogenase protein, which is homologous to the *E. coli* QorA, known to respond to oxidative stress as part of the electron transport chain (Edwards et al., 1996). Both the transcript and protein of *ZMO1993* were down-regulated in the acetate tolerant mutant AcR strain compared to the ZM4 strain (Yang et al., 2013). In *Clostridium thermocellum*, a mutation to alcohol dehydrogenase improved ethanol tolerance through changing NADH-dependent activity to NADPH-dependent, thus altering the electron transport chain in the mutant (Brown et al., 2011). These are consistent with our results that Zms4 promotes the degradation of *ZMO1993* transcript through its 5′-UTR, thereby also decrease the protein expression to improve the bacterial ethanol tolerance under stress. Interestingly, another confirmed Zms4 target, *ZMO1696*, also encodes an alcohol dehydrogenase family protein and interacts with Zms4 with high affinity. Even though we failed to discern the regulatory outcomes of Zms4 on this gene, the mutagenesis results showed that Zms4 binds to the *ZMO1696* mRNA through the 5′-UTR region (and through the Shine-Dalgarno (SD) region) (Figure 5A), which probably results instability of this transcript and/or reduced translation. *ZMO1696* is also previously shown to influence the glucose consumption and ethanol production in *Z. mobilis*, which may explain the elevated ethanol levels in the Zms4 overexpression strain (Figure 1B). Additionally, *ZMO1312* interacts with Zms4 but with a relatively low affinity (Figures 4A,B) and there is no clear clue about its contribution to the ethanol metabolism. Thus, we didn’t study this target in detail.

In terms of Zms6, *ZMO1934* is the strongest target and negatively regulated by Zms6 *in vivo* (Figure 6B). Interestingly, three binding sites responsible for Zms6–*ZMO1934* interaction were identified by mutagenesis analysis: all the three binding sites are important but only the 5′-UTR region is indispensable (Figure 5E and Supplementary Figure S5), which is supported by the *in vivo* results that Zms6 only influences the expression of the 5′-UTR but has little effect on the coding region (Figures 6B,D). Functionally, *ZMO1934* encodes the methyltransferase subunit of the type I restriction-modification system, which has been linked to lower glucose utilization rate (Kerr et al., 2011). Therefore, the repression of *ZMO1934* by Zms6 could be important in reducing DNA replication in order to conserve energy under ethanol stress but more likely to prevent the ethanol damaged/methylated DNA from entering the cell. The expression of the lysine exporter gene *ZMO1437* shows a lower affinity but is confirmed to be positively affected by Zms6 through the coding region (Figure 6D). The up-regulated expression of *ZMO1437* was previously shown to improve the tolerance to phenolic aldehydes (Yi et al., 2015), and Zms6 potentially stabilizes this transcript based on our results. Therefore, the negative regulation of *ZMO1934* and *ZMO1437* could improve the bacterial ethanol tolerance. However, it is intriguing that the *ZMO0170* level does not change in the Zms6 deletion strain (Figures 6B,D), even though this gene exhibits a high affinity to Zms6 *in vitro* (Figures 4C,D). One cannot be excluded that the Zms6 regulation on this target is not strong enough to detect due to the complex *in vivo* condition. Other factors, like Zms4, might also interfere with this regulation by competing the same region on Zms6 with a higher affinity where Zms6-*ZMO0170* base-pairing locates (Figure 4F).

Importantly, in this study, the co-regulation of various pathways by multiple sRNAs is especially insightful. Notably, we suspect that the mutual interaction between Zms4 and Zms6 leads to cross-talk regulatory effects on the targets. For instance, *ZMO1437*, the target upregulated by Zms6, is also positively influenced by Zms4 (Figure 6D). Some of the Zms4 targets, like *ZMO1993*, also display differential expression in the Zms6 deletion strain (Figure 6A). Interestingly, abolishing the expression of Zms4 does not affect transcript levels of Zms6 and vice versa (Figures 6E,F). Thus, we hypothesize that the Zms4–Zms6 interaction affects the binding between sRNAs and their targets through competition or structural changes. Furthermore, the interaction between Zms4 and Zms6 allows them to co-regulate a similar set of pathways important to ethanol stress response, especially the pathways related to translation, hydrogen sulfide biosynthetic process, sulfate assimilation, cysteine biosynthetic process and transport (Figures 2C,D). Nevertheless, several pathways were uniquely regulated by Zms6 overexpression, showing that Zms6 exhibits other regulatory roles instead of only serving as a Zms4 target.

*Second*, several other uncharacterized sRNAs are also involved in the Zms4–Zms6 regulatory network. In the MAPS assay, 16 other sRNAs were co-purified with Zms4 except for Zms6, whereas only two sRNAs (sRNA37 and Zms16) were pulled down with Zms6. In addition, our transcriptomic data also discovered a large number of sRNAs differentially expressed upon Zms4/Zms6 induction, including these directly bound sRNA targets (Supplementary Tables S2, S4). These changes might result from some regulators or RNA-binding proteins affected by Zms4 or Zms6 in an indirect way. Our previous study identified that the UTR region of *hfq* gene (ZMO0347) is sensitive to ethanol stress and act as a post-transcriptional regulator to increase sensitivity of Hfq protein under lower-level ethanol stress (Cho et al., 2017). In this study, the UTR region of *hfq* gene was found to be one of the targets co-purified with Zms4 (Figure 3 and Supplementary Table S4), but interaction between them was not detected in our study. These indicate that the regulation of Zms4 and Zms6 does not rely on the expression of Hfq, even though Hfq is confirmed to be essential for the fitness of *Z. mobilis* (Supplementary Figure S2C). However, our transcriptional analysis showed that both Zms4 and Zms6 positively regulate the expression of *hfq* gene as well as its 5′UTR region (Supplementary Table S2). This strongly indicates that these two sRNAs potentially affect a wide range of sRNAs through indirectly modulating Hfq expression.

*Moreover*, we identified the combinatorial effect of sRNAs on ethanol tolerance by overexpressing them simultaneously, based on the observation that some pathways are specifically regulated by Zms4 or Zms6. We selected Zms16 because it was confirmed to be one of the strongest targets of Zms6, and it is the only sRNA confirmed to directly interplay with Zms4/Zms6 in this study (Figures 4C,D). Our results showed that either single or combined sRNA overexpression strains exhibited different growth rate under ethanol stress (Supplementary Figure S7). Notably, the combination expression of all three sRNAs (pZms4-6-16) showed the highest growth rate under 6% ethanol. However, the benefits of sRNA expression are not always directly additive in this system as previously reported for three sRNAs involved in *E. coli* acid tolerance (Gaida et al., 2013). For example, the overexpression of single sRNAs (pZms4 and pZms6) showed decreased growth rate under ethanol, whereas the pZms4-6 strain shows the highest growth rate under no ethanol condition, likely due to some metabolic changes of harboring a plasmid overexpressing the two interacting sRNAs (albeit under the same P*_*tet*_* promoter) simultaneously. Therefore, this indicates that the network represents a potentially delicate interplay of sRNAs defined by optimal stoichiometric ratios.

**FIGURE 7:**
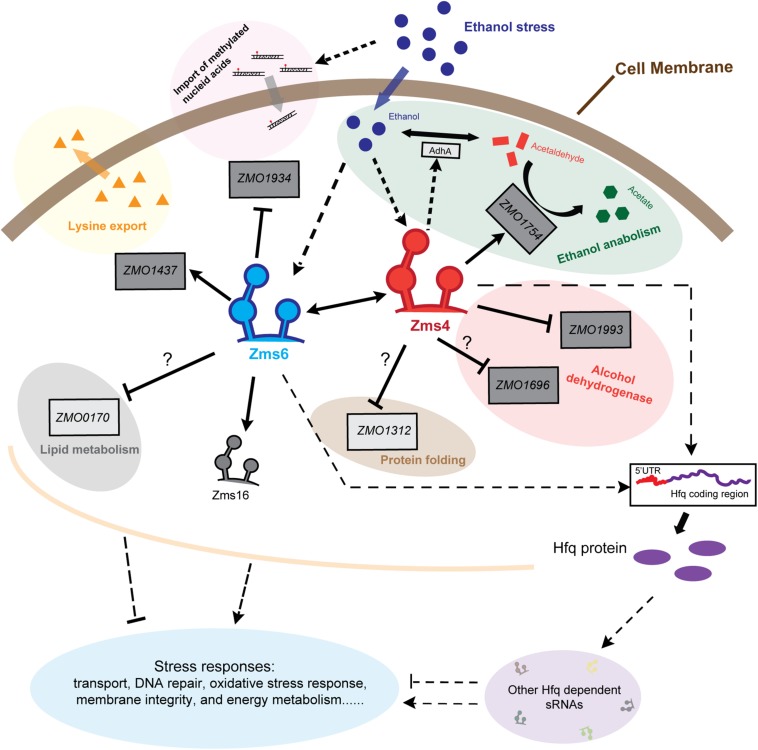
Schematic of Zms4–Zms6 regulatory networks involving sRNA interactions and direct mRNA targets in *Z. mobilis*. The accumulation of ethanol within the cells under stresses increase the expressions of Zms4 and Zms6. The increased Zms4 level accelerates the ethanol catabolism through upregulating aldehyde dehydrogenase gene (*ssdA*/*ZMO1754*) directly and alcohol dehydrogenase 1 gene (*adhA*) indirectly to covert ethanol to other carboxylic acids. Zms4 also affects electron transport and protein folding via directly binding to the 5′-UTR of alcohol dehydrogenase gene (*ZMO1993*) and peptidyl-prolyl *cis–trans* isomerase (*ZMO1312*). The other Zms4 target (*ZMO1696*), encoding a Zinc-binding alcohol dehydrogenase family protein, interacts with Zms4 through the SD region and potentially downregulated by Zms4. On the other hand, the upregulated Zms6 under ethanol stress upregulates the expression of lysine export gene *ZMO1437* and downregulates the expression of N-6 DNA methylase gene *ZMO1934* to improve the ethanol tolerance and to prevent the import of methylated DNA caused by ethanol damage, respectively. However, the regulation of Zms6 on *ZMO0170* is still a mystery since their binding doesn’t result in any gene expression change in this study. Solid lines in this figure represent the confirmed bindings/regulations and dotted lines represent the bindings/regulations that are not yet confirmed (arrows for upregulation, bars for downregulation). Dark gray boxes represent the confirmed targets and light gray boxes represent the targets that are not yet confirmed.

Our work thus far proposes a sRNA regulatory network in *Z. mobilis* (Figure 7), where two newly uncovered sRNAs, Zms4 and Zms6, interact with each other and collaboratively regulate several mRNA targets *in vivo* that affect ethanol resistance, probably through the competitive binding and/or structural changes. It is worth noting that only Zms4 and Zms6 were specifically characterized in this study, so additional pathways may also be affected given the likelihood that Zms16 and other sRNAs have their own separate targets. Overall, our results strongly suggest that the sRNA regulatory network in *Z. mobilis* is part of general stress response mechanisms, contributing to comprehensive effects on multiple pathways essential to the ethanol tolerance, such as transport, DNA repair, ethanol anabolism, and energy metabolism.

Taken together, this concept of a sRNA–sRNA interaction regulating bacterial tolerance and fitness under ethanol stress as seen here in *Z. mobilis* is novel and we expect to see the emergence of similar networks in other bacterial species to regulate other complex phenotypes.

## Data Availability Statement

High-throughput sequencing datasets have been deposited in NCBI GEO under accession number GSE107219.

## Author Contributions

LC, RH, and KH conceived and designed the experiments. RH, KH, JG-R, SC, and BS performed the experiments and analyzed the data. SY, YY, RL, and JH conceived and conducted the *in vivo* validation of the sRNA targets using the reporter systems. LC, RH, and KH wrote and revised the manuscript.

## Conflict of Interest

The authors declare that the research was conducted in the absence of any commercial or financial relationships that could be construed as a potential conflict of interest.
