# Supplementary material for: Multiple Small RNAs Interact to Co-regulate Ethanol Tolerance in Zymomonas mobilis
Source: Front Bioeng Biotechnol. 2020 Mar 4;8:155. doi: 10.3389/fbioe.2020.00155 (PMC7064620; doi:10.3389/fbioe.2020.00155)

Supplementary Material

**Multiple small RNAs interact to co-regulate ethanol tolerance in *Zymomonas mobilis***

**Runhua Han^1†^, Katie Haning^1†^, Juan C. Gonzalez-Rivera^1^, Yongfu Yang^2^, Runxia Li^2^, Seung Hee Cho^3^, Ju Huang^2^, Bobi A. Simonsen^1^, Shihui Yang^2^, Lydia M. Contreras^1,3*^**

^1^ McKetta Department of Chemical Engineering, The University of Texas at Austin, Austin, TX, 78712, USA

^2^ State Key Laboratory of Biocatalysis and Enzyme Engineering, Hubei Collaborative Innovation Center for Green Transformation of Bio-resources, Environmental Microbial Technology Center of Hubei Province, and School of Life Sciences, Hubei University, Wuhan, 430062, China

^3^ Institute for Cellular & Molecular Biology, The University of Texas at Austin, Austin, TX, 78712, USA

^†^These authors contributed equally to this work

*** Correspondence:**Lydia M. Contreras
lcontrer@che.utexas.edu

**LIST OF SUPPLEMENTARY INFORMATION**

**Supplementary Figures**

**Figure S1**. Verification of Zms4/Zms6 levels in sRNA deletion and overexpression strains.

**Figure S2**. The regulation of Hfq on Zms4 and Zms6.

**Figure S3**. Detection of Zms4 and Zms6 *in vitro* interactions with all tested RNA targets by EMSA.

**Figure S4**. Predicted binding sites of confirmed targets of Zms4 and Zms6 by IntaRNA.

**Figure S5**. Affinities of RNA-RNA complex formed between either Zms6 and ZMO1934 as well as its three mutant forms (ZMO1934mut1, ZMO1934mut2 and ZMO1934mut3).

**Figure S6**. *In vivo* reporter systems developed for detecting sRNA regulatory outcomes on mRNA targets through the binding sites.

**Figure S7**. Combined sRNA overexpression indicates complexity of the network.

**Supplementary Tables**

**Table S1**. Strains and plasmid used in this study.

**Table S2**. Primers used in this study.

**Table S3**. Transcripts significantly dependent on Zms4 and Zms6.

**Table S4**. Transcripts co-immunoprecipitated with Zms4 and Zms6.

**Table S5**. Proteins co-immunoprecipitated with Zms4 and Zms6.

**SUPPLEMENTARY FIGURES**

**Figure S1**

Verification of transcript levels of Zms4 and Zms6 in sRNA deletion and overexpression strains. Northern blotting shows successful deletion of Zms4 (**A**) and Zms6 (**B**). Transcript counts (normalized by DESeq2) show inducible overexpression of Zms4 (**C**) and Zms6 (**D**) over time. Each point represents a biological duplicate and the line their average.


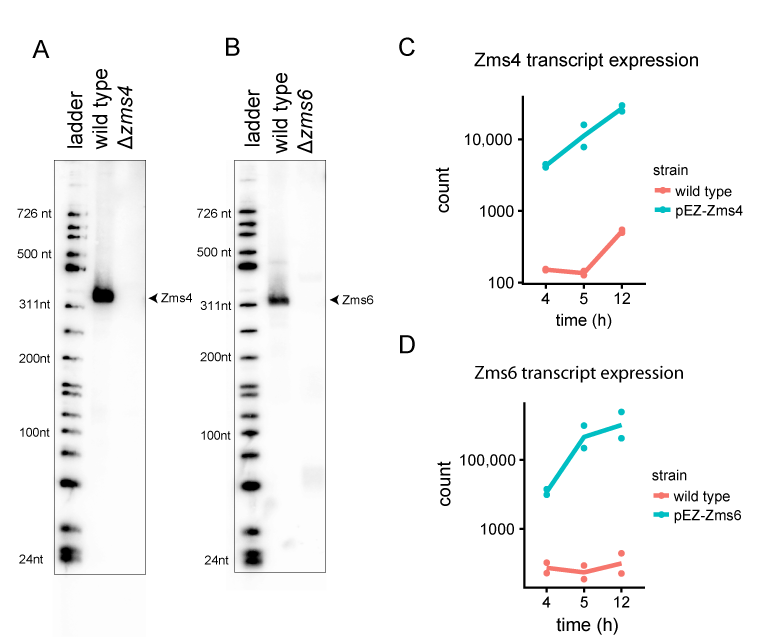


**Figure S2**

The regulation of Hfq on Zms4 and Zms6. EMSA shows that there is no interaction between Hfq and Zms4/Zms6 (**A**). Northern blotting shows the deletion of *hfq* gene doesn’t affect the expression of Zms4 and Zms6 (**B**). The expression of *hfq* significantly affect the growth of *Z. mobilis* (**C**).


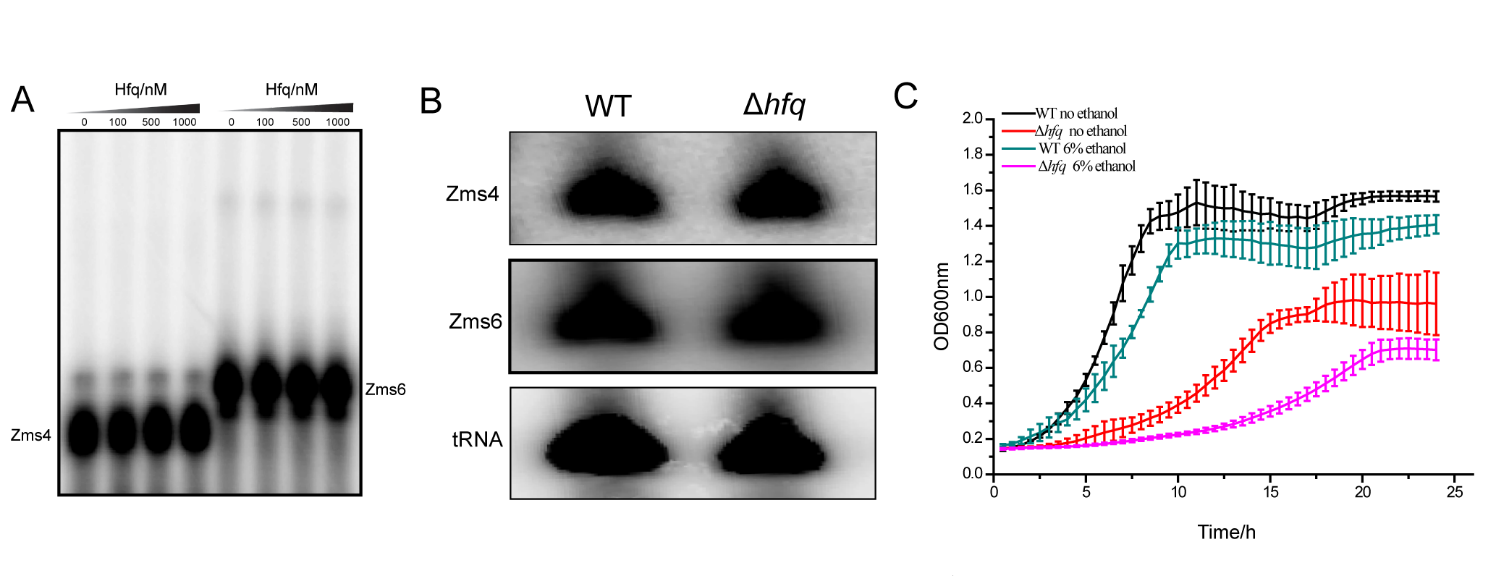


**Figure S3**

Detection of Zms4 and Zms6 *in vitro* interactions with all RNA targets by EMSA. All pairs are tested by incubating 5 pmol radiolabeled Zms4 (**A**) or Zms6 (**B**) with 100 pmol target and those with a band shift with various affinities (indicating a confirmed *in vitro* interaction) are also shown in **Figure 4A** and **4C**.


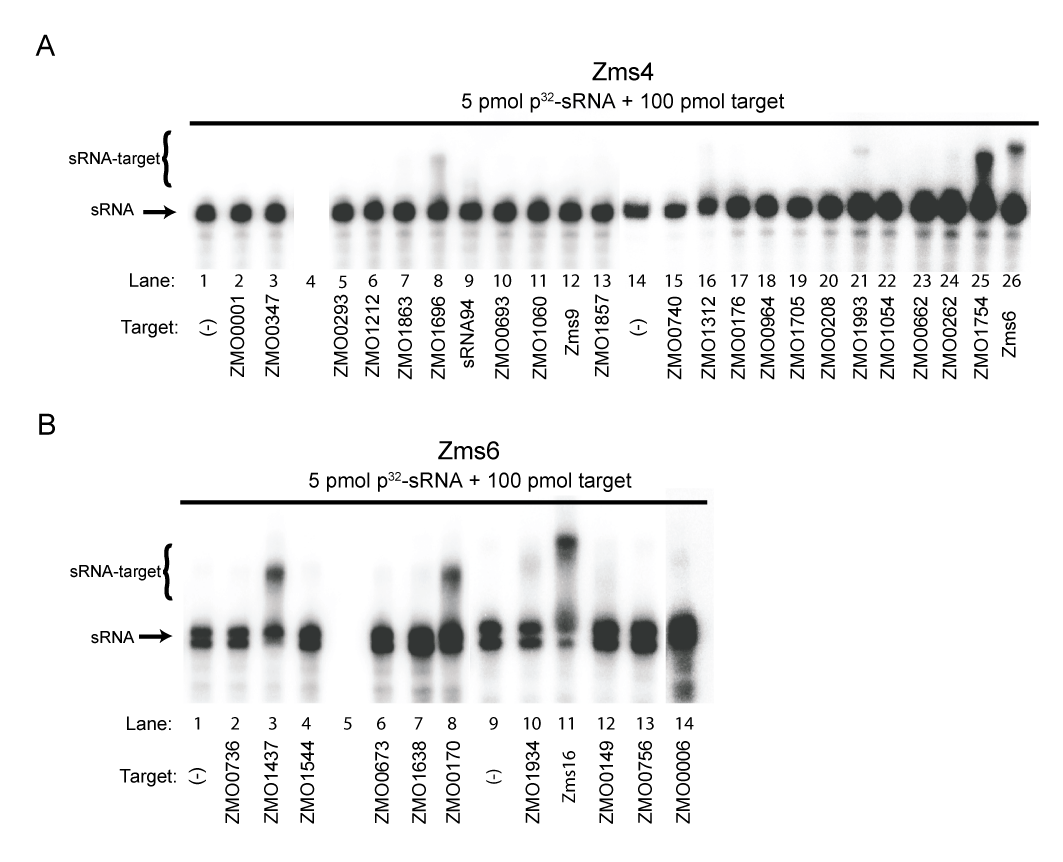


**Figure S4**

Predicted binding sites of confirmed targets of Zms4 (**A**) and Zms6 (**B**) by IntaRNA. The start codon (AUG) is highlighted by blue.


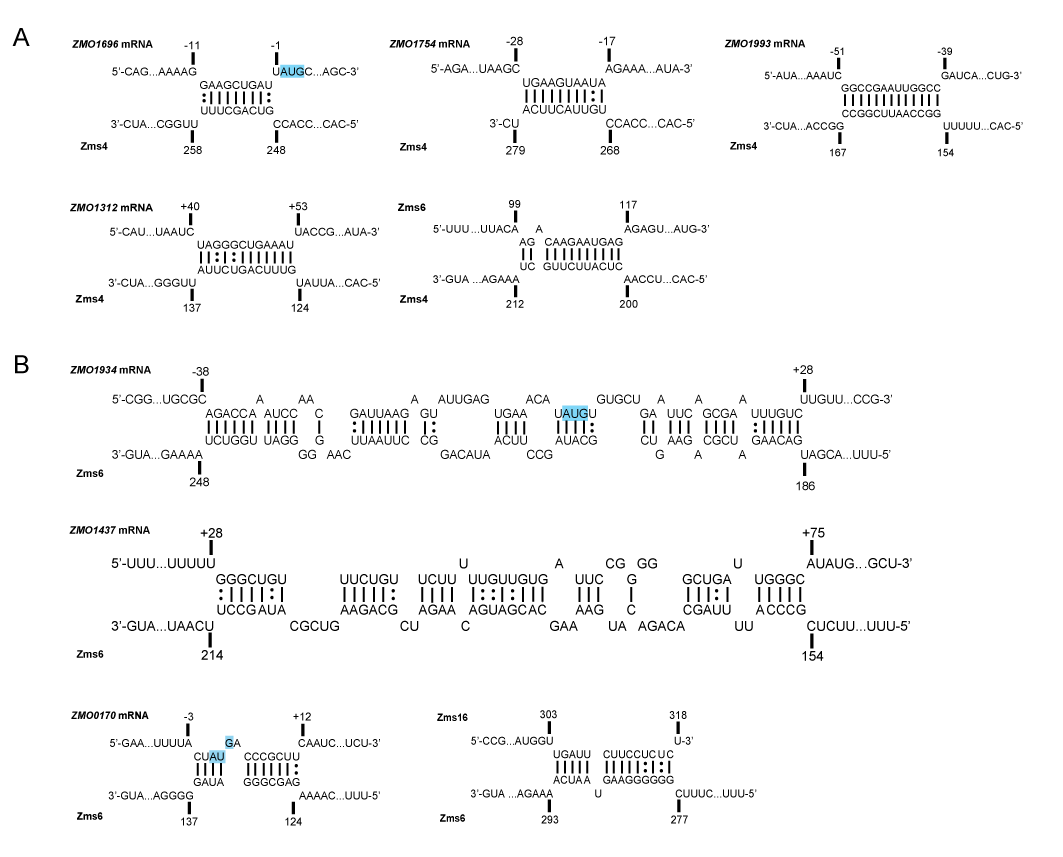


**Figure S5**

Affinities of RNA-RNA complex formed between Zms6 and *ZMO1934* as well as its three mutant forms (*ZMO1934*mut1, *ZMO1934*mut2 and *ZMO1934*mut3).


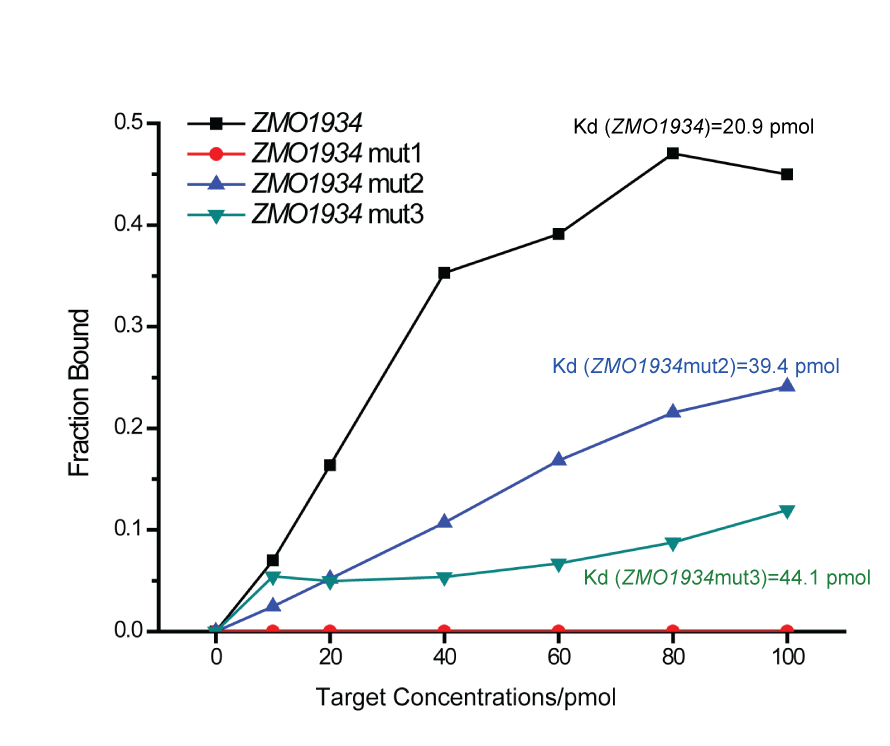


**Figure S6**

*In vivo* reporter systems developed for detecting sRNA regulatory outcomes on mRNA targets through the binding sites. The sRNA regulation of the 5’UTR was detected using the Dual-reporter system (**A**). The 5’ region (-200 nt to +99 nt) of each mRNA target was examined for predicted promoters by BPROM. The predicted transcription starts site (TSS) was used as the start site for amplification and cloning of each 5’ UTR into a dual fluorescence expression vector. The 5’ UTR + *EGFP* fusion was expressed under the constitutive P*_gap_* promoter. As a control *mCherry* is expressed by PlacUV5. Each vector was transformed into the wild type 8b strain and sRNA deletion strains of its corresponding sRNA. The sRNA regulation of the coding regions was detected using the promoter replacement system (**B**). The native promoter of the target gene was replaced with the *tetA* promoter and *mCherry* gene by homologous recombination, and the *tetA* promoter initiates co-transcription of *mCherry* and target gene. The Zms4 and Zms6 overexpression plasmids were then introduced to these strains using the pEZ-tet vector with kanamycin gene replacing the original spectinomycin gene (pEZ-Kana). The empty vector was also transformed into these strains as the control.


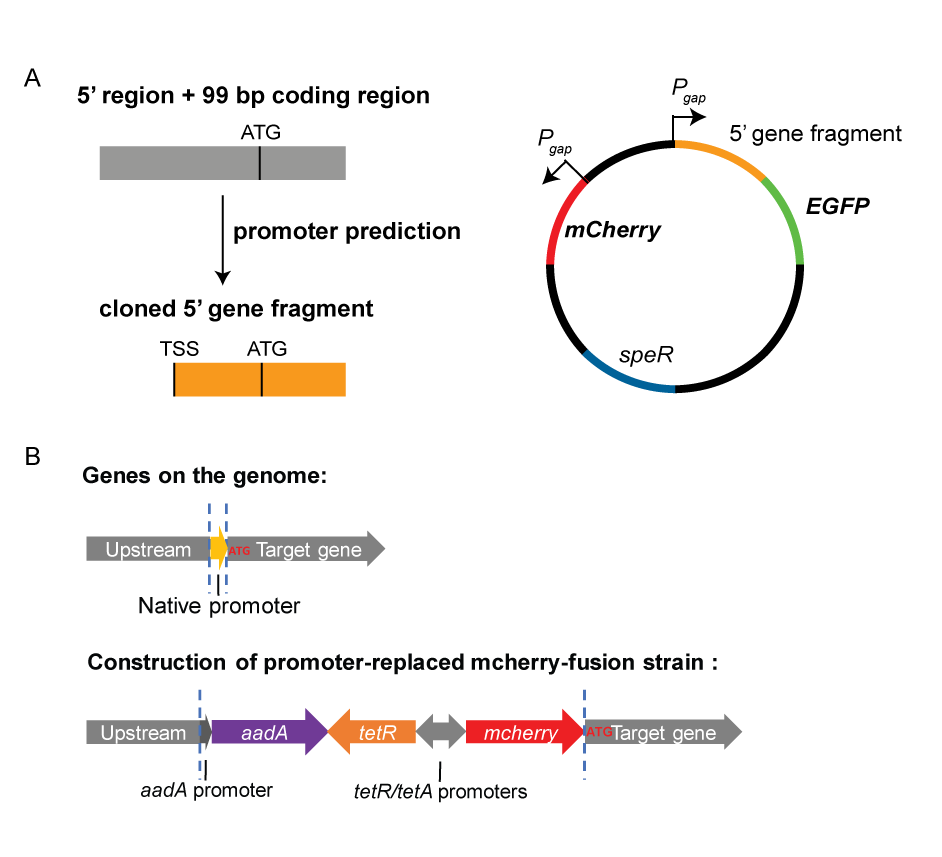


**Figure S7**

Combined and single sRNA overexpression indicates complexity of the network. Wild type with empty vector and overexpression strains were grown anaerobically with and without 6% (v/v) ethanol supplementation. The turbidity (600 nm) was measured every 0.5 h. Growth curves of all the strains under no ethanol (**A**) and growth curves of all the strains under 6% ethanol (**B**) are shown.


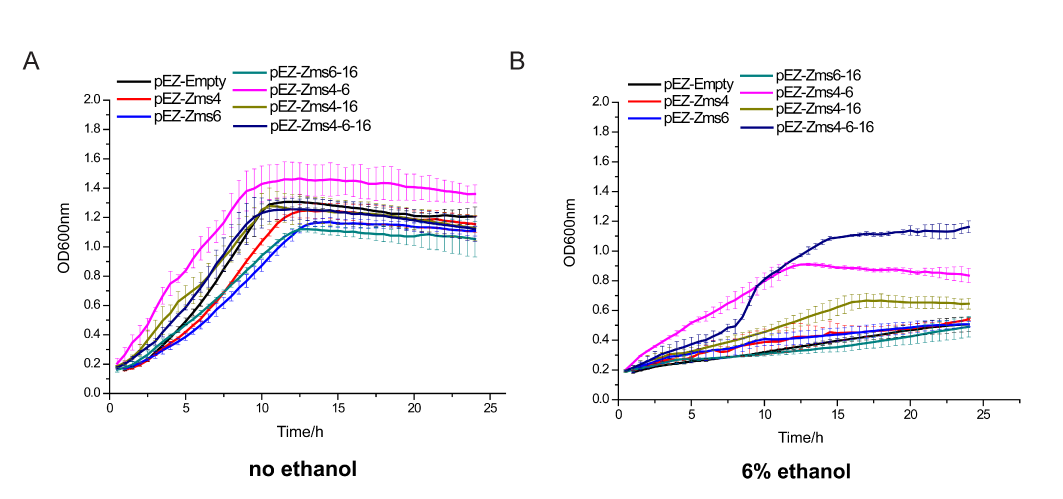

Supplement: Supplementary file 2 [file Table_2.docx]
